# Development of a high‐resolution two‐dimensional detector‐based dose verification system for tumor‐tracking irradiation in the CyberKnife system

**DOI:** 10.1002/acm2.13645

**Published:** 2022-07-05

**Authors:** Fumitaka Kawabata, Takeshi Kamomae, Kuniyasu Okudaira, Masataka Komori, Hiroshi Oguchi, Motoharu Sasaki, Masaki Mori, Mariko Kawamura, Shinji Abe, Shunichi Ishihara, Shinji Naganawa

**Affiliations:** ^1^ Department of Radiological Technology Nagoya University Hospital Nagoya Aichi Japan; ^2^ Department of Radiology Nagoya University Graduate School of Medicine Nagoya Aichi Japan; ^3^ Department of Integrated Health Sciences Nagoya University Graduate School of Medicine Nagoya Aichi Japan; ^4^ Department of Therapeutic Radiology Institute of Biomedical Sciences Tokushima University Graduate School Tokushima Japan

**Keywords:** basic characterization, CyberKnife, SRS MapCHECK, tumor‐tracking

## Abstract

We aim to evaluate the basic characteristics of SRS MapCHECK (SRSMC) for CyberKnife (CK) and establish a dose verification system using SRSMC for the tumor‐tracking irradiation for CK. The field size and angular dependence of SRSMC were evaluated for basic characterization. The output factors (OPFs) and absolute doses measured by SRSMC were compared with those measured using microDiamond and microchamber detectors and those calculated by the treatment planning system (TPS). The angular dependence was evaluated by comparing the SRSMC with a microchamber. The tumor‐tracking dose verification system consists of SRSMC and a moving platform. The doses measured using SRSMC were compared with the doses measured using a microchamber and radiochromic film. The OPFs and absolute doses of SRSMC were within ±3.0% error for almost all field sizes, and the angular dependence was within ±2.0% for all incidence angles. The absolute dose errors between SRSMC and TPS tended to increase when the field size was smaller than 10 mm. The absolute doses of the tumor‐tracking irradiation measured using SRSMC and those measured using a microchamber agreed within 1.0%, and the gamma pass rates of SRSMC in comparison with those of the radiochromic film were greater than 95%. The basic characteristics of SRSMC for CK presented acceptable results for clinical use. The results of the tumor‐tracking dose verification system realized using SRSMC were equivalent to those of conventional methods, and this system is expected to contribute toward improving the efficiency of quality control in many facilities.

## 1 INTRODUCTION

Stereotactic body radiotherapy (SBRT) is a widely used treatment option for lung or liver cancer patients, and highly effective local tumor control rates with minimal side effects have been reported.^1^, ^2^, ^3^, ^4^, ^5^ Recent advances in radiation therapy treatment systems, such as the development of a high‐precision linear accelerator (linac) with the multileaf collimator (MLC), treatment planning systems (TPSs), computed tomography (CT) scanners for treatment planning, and image guidance systems, have contributed to improved clinical outcomes.^6^, ^7^, ^8^ In addition to these advances, quality assurance and quality control for clinical equipment have generally been recognized as essential and standard practices.^9^

The CyberKnife radiotherapy system (CK; Accuray Inc., Sunnyvale, CA, USA) is specifically designed for stereotactic radiation therapy and is composed of a robotic manipulator, a small linear accelerator, a six‐dimensional robotic couch, and an image‐guidance system. It can irradiate a target from various directions around a patient while maintaining high‐accuracy target localization through imaging and positional correction during treatment.^10^ The synchrony respiratory tracking system (SRTS) provides dynamic tumor‐tracking irradiation using implanted fiducial markers with optical markers placed on the body of a patient. It is used to treat organs that exhibit respiratory motion, such as the lungs and liver. Using a tumor‐tracking method such as SRTS, it is possible to reduce the radiation dose to normal tissues around a target with respiratory motion compared to other irradiation techniques such as the free‐breathing method.^11^ Moreover, guidelines for radiation therapy on organs with respiratory motion have been published, and the importance of patient dose verification in tumor‐tracking irradiation has been described. Especially, the importance of positional accuracy in dose distribution verification has been established.^12^

Recently, the Sun Nuclear Corporation (Melbourne, FL, USA) released the SRS MapCHECK (SRSMC) diode array. SRSMC is composed of 0.48 × 0.48 mm^2^ diode detectors, with a size smaller than the recommended 1.0‐mm maximum detector size for use in patient dose verification systems identified in the guidelines for SBRT.^13^ Moreover, it has a higher resolution than conventional two‐dimensional array detectors.^14^ Therefore, it is suitable for small field measurements such as stereotactic radiosurgery and stereotactic irradiation. Ahmed et al.^15^ reported the basic characterization of SRSMC in a general‐purpose linac. The field size dependence showed that the output factor (OPF) disagreement for the scintillator detector was up to 3.2% in irradiation fields of 10 × 10 to 40 × 40 mm^2^, and the angular dependence showed that the response deference was within 2.0% for ±90° gantry angles, except when a parallel beam was incident on the detector. In addition, the monitor unit (MU) value linearity and repetition rate dependence agreed within 2.0% with respect to the ionization chamber. Subsequently, Rose et al.^16^ reported the results of a multi‐institution study on patient dose verification using SRSMC and showed that the obtained measurement results were equivalent to those achieved using film dosimetry.

By contrast, the characterization of SRSMC for CK has not yet been reported. Unlike the general‐purpose linac, CK uses many extremely small fields, irradiates from multiple directions with a robotic manipulator, and tracks a moving target with SRTS. When considering the use of SRSMC with CK, it is necessary to evaluate the unique characteristics of this detector, such as the field size dependence and angular dependence for CK, in addition to the previous results reported for the general‐purpose linac.

Moreover, the quality control of the tumor‐tracking irradiation for CK is generally performed by combining a radiochromic film and a moving phantom, as reported by the American Association of Physicists in Medicine Task Group 135.^17^ Akino et al. and Yang et al. proposed verification methods for tumor‐tracking irradiation for CK without using films. Akino et al.^18^ proposed a verification system combining a plastic scintillator with a U‐shaped plastic frame and evaluated the motion tracking accuracy of the SRTS. Yang et al.^19^ proposed a tracking accuracy verification method using a moving phantom and a two‐dimensional detector array (OCTAVIUS Detector 1500; PTW Freiburg, Germany). These methods can verify the tracking accuracy,^18^, ^19^, ^20^ but it is necessary to perform patient‐specific dose verification such as that for the absolute dose and dose distribution during tumor‐tracking by SRTS. To the best of our knowledge, geometric and dosimetric verifications for tumor‐tracking irradiation for CK with a 2D (or 3D) detector have not been reported, except for films.

The purpose of this study is to evaluate the basic dosimetric characterizations of SRSMC for CK and to establish a filmless patient‐specific dose verification system using SRSMC for tumor‐tracking irradiation by SRTS in CK. Our proposed system is composed of SRSMC, the StereoPHAN phantom, and a commercial moving platform without any additional equipment and could be realized at any facility having access to this equipment.

## 2 MATERIALS AND METHODS

### 2.1 Details of SRSMC

**FIGURE 1:**
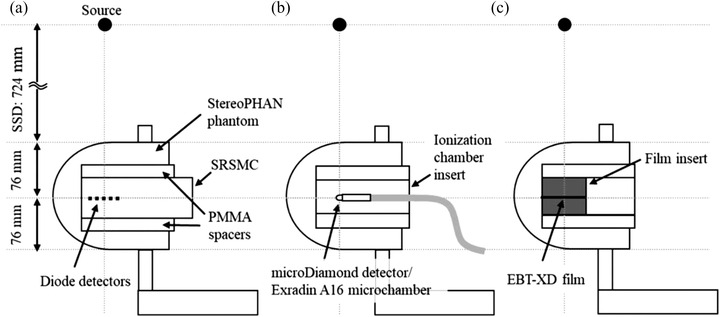
Schematic of the StereoPHAN phantom with SRS MapCHECK (SRSMC), the ionization chamber insert, and the film insert: (a and b) measurement geometries for the field size and angle dependence evaluations and (a–c) measurement geometries for the dose verification of the tumor‐tracking irradiation system. The detector surface of the SRSMC inserted in the StereoPHAN agreed with the detector's active volume centers of the PTW microDiamond detector, Exradin A16 microchamber, and EBT‐XD film plane

A total of 1013 diode detectors with an active volume of 0.007 mm^3^ (0.48 × 0.48 mm^2^ cross section) are arranged in the SRSMC detector plane with an area of 77 × 77 mm^2^. The distance between the diode centers is 2.47 mm. The SRSMC body is composed of poly(methyl methacrylate) (PMMA), and a circuit board is installed on the caudal side. SRSMC is designed for use with the StereoPHAN phantom (Sun Nuclear Corp., Melbourne, FL, USA) with 22‐mm‐thick PMMA spacers placed at the top and bottom of the SRSMC. The measured data are analyzed using the SNC Patient software (Ver. 8.3; Sun Nuclear Corp., Melbourne, FL, USA), and four correction factors (i.e., the field size, angle, temperature, and repetition rate) can be applied selectively during measurements. In this study, measurements were performed with all corrections applied. In addition, by including an ionization chamber or film inserts (Sun Nuclear Corp., Melbourne, FL, USA) with appropriate spacers into the StereoPHAN phantom, measurements can be performed at the same measurement point or plane as in SRSMC. The fiducial markers in SRSMC, film insert, or universal spacer insert are used for phantom positioning. A schematic of the StereoPHAN phantom with the detectors is shown in Figure 1.

### 2.2 Field size dependence of SRSMC for CK

The field size dependence of SRSMC for CK was evaluated using two factors: (1) the OPF for basic physical characterization and (2) the absolute dose. Prior to the experiments, the uniformity of the SRSMC detectors was calibrated according to the method specified by the manufacturer (Sun Nuclear Corp., Melbourne, FL, USA).^21^ After calibration, CK was arranged at a source axis distance (SAD) of 800 mm to irradiate a beam perpendicular to the detector plane (Figure 1a). The irradiation fields were formed using fixed circular collimators, and the field diameters were changed from 5 to 60 mm in 12 steps. The parameters for the beam irradiation were an MU of 200, a dose rate of 1000 MU/min, and a 6‐MV flattening filter free beam. The OPFs were calculated by normalizing the response of the diode detector located at the center of the SRSMC detection area in each field of the fixed circular collimator with a response at a diameter of 60 mm. The OPFs of the SRSMC were compared with those measured using a microDiamond detector (model 60019; PTW Freiburg, Germany). The measurement geometry of the microDiamond detector is shown in Figure 1b. The OPFs of the microDiamond detector were estimated using either the raw data or by applying the field output correction factors provided in International Atomic Energy Agency (IAEA) Technical Report Series No. 483 (TRS‐483).^22^

Subsequently, absolute dose evaluation was performed by comparing the doses measured by SRSMC and the Exradin A16 microchamber (Standard Imaging., Madison, WI, USA), as well as that calculated using the Precision TPS (Ver. 2.0.1.1; Accuracy Inc., Sunnyvale, CA, USA). The field output correction factors in IAEA TRS‐483 were applied to the dose measured using the Exradin A16 microchamber as well as the OPF evaluation. TRS‐483 does not recommend a detector‐specific output correction factor greater than ±5.0%; therefore, a field output correction factor of 5‐mm field size for the Exradin A16 microchamber was not applied in this study. The details of each absolute dose calculation are shown below. According to the method recommended by the manufacturer (Sun Nuclear Corp., Melbourne, FL, USA), the absolute dose of SRSMC was calibrated using the following procedure. Prior to the calibration, SRSMC and StereoPHAN were imaged using a Somatom Confidence RT Pro CT scanner (Siemens Healthineers., Forchheim, Germany). The scanning parameters were a tube voltage of 120 kV, field of view of 500 mm, and slice thickness of 1.0 mm. The CT images were then imported into the TPS, and the CT number in the StereoPHAN region was overwritten using the following procedure. First, the CT number corresponding to the mass density of 1.2 g/cm^3^ recommended by the manufacturer (Sun Nuclear Corp., Melbourne, FL, USA) was estimated using the CT number to mass density conversion curve at our institute. The relative electron density corresponding to the CT number was then calculated using the relative density conversion curve. Finally, the StereoPHAN region was overwritten with a relative electron density of 1.15. The treatment plan for a single beam with a 54.6 × 54.6 mm^2^ field using the MLC was created utilizing the TPS. The irradiation MU was 100 MU, and the SAD was 800 mm. The selectable dose calculation algorithms when using MLC are finite‐size pencil beam (FSPB), where FSPB is with lateral scaling correction (FSPB+), and Monte Carlo (MC).^7^ When MC is selected for dose calculation, the CT numbers are inevitability assigned to three materials, that is, air (mass density: <0.1 g/cm^3^), soft‐tissue (0.1–1.125 g/cm^3^), and bone (>1.125 g/cm^3^), to define the mean free path length of a photon.^23^ Therefore, the MC implemented in Precision TPS cannot calculate the dose on the overwritten CT number. To reduce complexity, FSPB+ was selected for dose calculations. The dose calculations were performed using FSPB+, and the dose of the diode detector located at the center of the SRSMC detection area was calculated. The dose calculations were performed at a high resolution with a calculation voxel size of 0.98 × 0.98 × 1.00 mm^3^. Absolute dose calibration for SRSMC was performed by registering the calculated dose of the diode detector located at the center of the SRSMC detection area using the SNC Patient software.

The absolute dose of the Exradin A16 microchamber cannot be directly derived because the fluence scaling and depth scaling corrections are not adaptable^24^ owing to the round outward form of the StereoPHAN phantom. In this study, the absolute doses for the Exradin A16 microchamber with a fixed circular collimator size *m* (mm) were calculated using the following equation:

(1) $$D_{A16,m}=M_{A16,m}\left(D_{TPS,60}/M_{A16,60}\right),$$

where *M*_A16, 60_ and *M*_A16,_ *_m_* are the fully corrected electrometer readings in coulombs (C) measured with a fixed circular collimator of 60 mm and *m*, respectively. *D*_TPS, 60_ is the dose calculated using the TPS under the same geometry and irradiated parameters. The dose calculation algorithms implemented in the TPS that can be selected when using a fixed circular collimator are ray tracing and MC. We selected ray tracing for the same reason described earlier.

Finally, SRSMC or the Exradin A16 microchamber inserted into the StereoPHAN phantom were irradiated under the same conditions used for the OPF evaluation, and the measured doses were compared with the doses calculated using the TPS. The OPF and absolute dose measurements were performed five times. These calculations were performed using the ray tracing algorithm under the condition that the StereoPHAN region was overwritten with a relative electron density of 1.15.

### 2.3 Angular dependence of SRSMC for CK

We used 113 single‐beam irradiation treatment plans from 113 nodes in the CK body path on the CT images of the StereoPHAN phantom with SRSMC using a fixed circular collimator with a diameter of 30 mm in the TPS. These plans were calculated using the ray tracing algorithm; the calculation parameters were the same as those in the experiment described in Section 2.2, and the irradiation MU was 200 MU. The created treatment plans were used to irradiate SRSMC and measure the absolute dose of the central diode detector. After this process, the treatment plans were created based on the CT images of the StereoPHAN phantom with a chamber insert for the Exradin A16 microchamber, and dose measurements were performed to determine the angular dependence at each node position by comparing the measured doses of SRSMC and those of the Exradin A16 microchamber.

### 2.4 Overview of the high‐resolution 2D detector‐based dose verification system for tumor‐tracking irradiation

**FIGURE 2:**
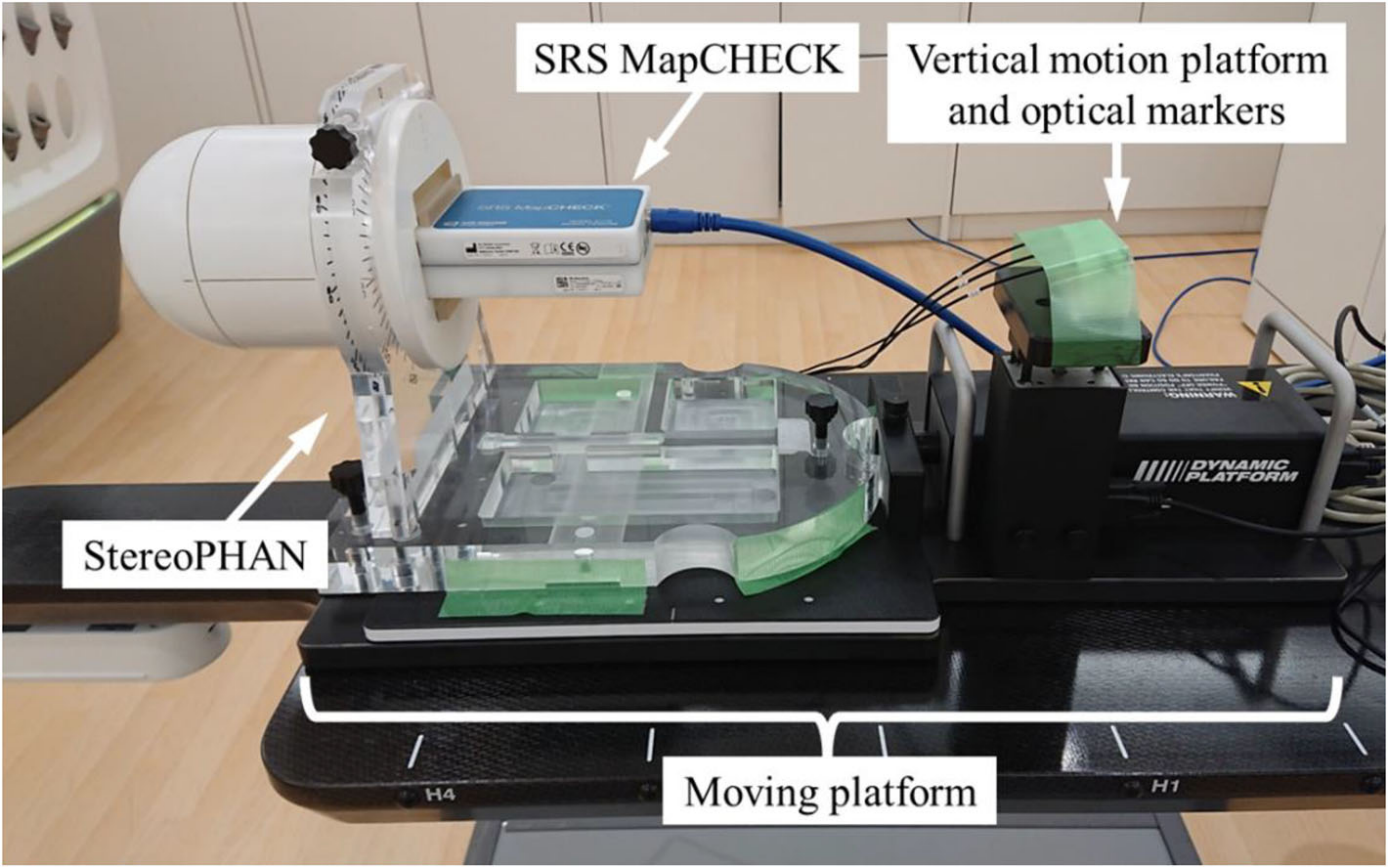
Overview of the high‐resolution 2D detector‐based dose verification system for tumor‐tracking irradiation

Figure 2 shows the proposed tumor‐tracking dose verification system using SRSMC. The tumor‐tracking system is composed of SRSMC, the StereoPHAN phantom, and a moving platform (Model 008PL; CIRS Inc., Norfolk, VA, USA). The maximum load indicated by the manufacturer of the moving platform is 32 kg, and the weights of SRSMC and StereoPHAN are within this specification. The moving platform has a motion accuracy of ±0.1 mm,^25^ and the base plate can be moved linearly up to a distance of 50 mm in the superior–inferior (S–I) direction using dedicated software. Optical markers were placed on a vertically moving platform to simulate the abdominal wall movements of patients.

### 2.5 Treatment plans for the verification of the tumor‐tracking irradiation

The treatment plans for the verification of the tumor‐tracking irradiation system were prepared using the same method reported by Kawata et al.^7^ A moving thorax phantom (Model 008A; CIRS Inc., Norfolk, VA, USA) and a water‐equivalent mock tumor with a diameter of 20 mm were used as a dummy patient. Table 1 shows the moving conditions of the phantom during CT imaging. The following waveform model was used for the wave function^8^, ^26^:

(2) $$A\;\left(t\right)=A_{0}\;-\left\{1-2\mathrm{co}s^{6}\left(\pi t/T-\emptyset \right)\right\}\times A/2,$$

where A(t) is the amplitude at time *t*, *A*_0_ is the reference position, and ∅ is the initial phase. The phantom under static condition was defined as P0, and the three respiratory motion conditions were defined as P1, P2, and P3. The scanning parameters were the same as those described in Section 2.2. The gross tumor volume was specified, and an isotropic margin of 5 mm was defined as the planning target volume (PTV). The treatment plan was created based on the specifications of the Japan Clinical Oncology Group 1408.^27^ Fractional doses of 13.5 Gy were prescribed to the 80% isodose line, with the dose covering 95% of the PTV. All treatment plans were created using MLC, and dose calculations were performed using the MC algorithm. A relative statistical uncertainty of 1.0% in the maximum dose point for the dose summed from all beams was set for the MC dose calculation, and the results were smoothed with a normalized Gaussian broadening width of 0.6. These created plans were converted to verification plans for the StereoPHAN phantom with SRSMC, the Exradin A16 microchamber, or the film. For tracking, fiducial tracking was selected for the phantom under static conditions, and SRTS was selected for the phantom under respiratory motion conditions. FSPB+ and MC were selected as the dose calculation algorithms. The calculation parameters of FSPB+ and MC were the same as those mentioned in Section 2.2 and described earlier. The phantoms were overwritten with a relative electron density of 1.15 when using FSPB+, and the manufacturer's recommended mass density of 1.2 g/cm^3^ was used for MC.

**TABLE 1:** Summary of the phantom motion conditions

		Respiratory motion condition		
Plan	Waveform	Respiratory cycle (s)	Amplitude (mm)	Movement direction
P0	Static			
P1	(cos)^6^	2	13	S–I
P2	(cos)^6^	4	25	S–I
P3	(cos)^6^	8	40	S–I

### 2.6 Data acquisition and analysis

The SRSMC and StereoPHAN were irradiated according to the created verification plans. The markers inserted in SRSMC were used for image matching during positioning. The results of the SRSMC measurements were compared with the doses calculated by the TPS using the SNC Patient software. The SRSMC measurement results were also compared with those measured using conventional verification methods such as an ionization chamber or a film.

Considering the conventional verification methods, the ionization chamber or film insert in the StereoPHAN phantom was placed on the moving platform as in SRSMC (Figure 1b,c). An Exradin A16 microchamber was used as the ionization chamber, and the formalism of the absorbed dose estimations was the same as that described in Section 2.2. Film measurements were conducted using a GAFchromic EBT‐XD film (Ashland ISP Advanced Materials, NJ, USA). Prior to the measurements, a dose calibration curve was created in the range of 0–30 Gy. Film pieces of 60 × 60 mm^2^ were placed in the center of the Solid Water HE slab phantom (Sun Nuclear Corp., Melbourne, FL, USA) at a SAD of 800 mm and a depth of 100 mm and irradiated using a 60‐mm fixed circular collimator. After 24 h, the irradiated films were scanned using the EPSON EXPRESSION 10000XL scanner (Epson Corp., Nagano, Japan) to generate 48‐bit color images with a resolution of 75 dots per inch. A dose calibration curve was created from the irradiated films in the SNC Patient software (Ver. 6.2.3; Sun Nuclear Corp., Melbourne, FL, USA). The films for tracking irradiation were cut into 75 × 75 mm^2^ pieces and inserted into the film insert of the StereoPHAN phantom for irradiation. Film scanning was performed in the same manner as for the preparation of the dose calibration curve.

The dose distributions measured by SRSMC were compared with those calculated using the TPS and those measured using a film with the SNC Patient software. Furthermore, position matching for the dose distribution analyses was performed using two methods: absolute position matching by marker alignment and relative position matching, which maximizes the pass rate for gamma analysis. The global gamma analysis was performed at a threshold of 10%, with dose difference and distance to agreement criteria of 3%/1 mm.

## 3 RESULTS

### 3.1 Field size dependence

**FIGURE 3:**
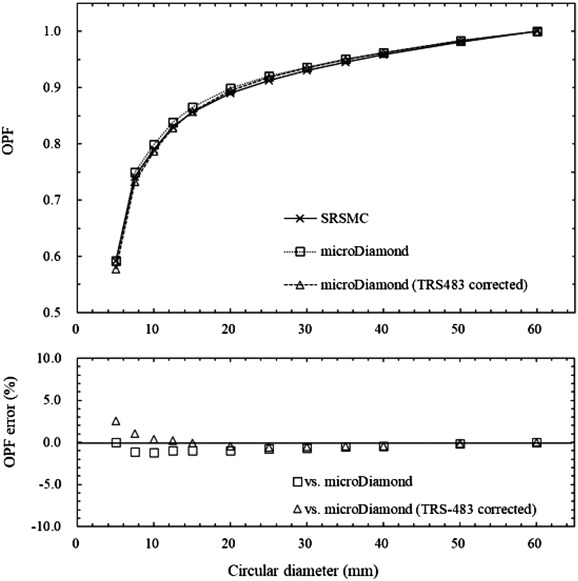
Comparison of the output factors (OPFs) measured by SRS MapCHECK (SRSMC), the microDiamond detector, and the microDiamond detector after the field output correction factors of TRS‐483 were applied. The upper graph shows the OPF measurement results, and the lower graph shows the percentage errors between the OPF measured by SRSMC and those measured by the microDiamond detector and the microDiamond detector after TRS‐483 corrections. Each measured value had an extremely small error range; thus we omitted error bars from the plots

Figure 3 shows a comparison of the OPFs measured by SRSMC, the microDiamond detector, and the microDiamond detector with the applied field output correction factors of TRS‐483. The largest OPF error between SRSMC and microDiamond was −1.2% and decreased to 0.4% when TRS‐483 corrections were applied. The OPF error between SRSMC and microDiamond were larger than 1.0% for the field sizes of 7.5–12.5 mm, but it was within 1.0% after the TRS‐483 corrections, except for the field sizes of 5–7.5 mm.

**FIGURE 4:**
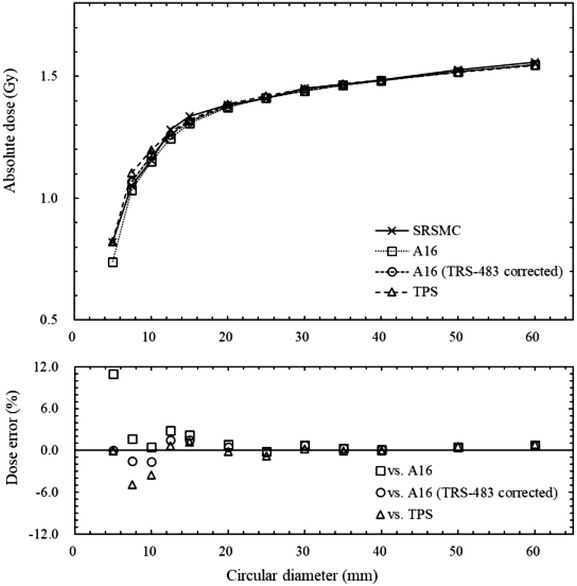
Comparison of the absolute dose measured by SRS MapCHECK (SRSMC), the Exradin A16 microchamber, the Exradin A16 microchamber with the applied field output correction factors of TRS‐483, and the dose calculated by treatment planning system (TPS). The bottom graph shows the measured dose error of the SRSMC relative to the measured doses of the Exradin A16 microchamber, Exradin A16 microchamber after TRS‐483 corrections, and calculated dose. Error bars are omitted because the error range is small

Figure 4 shows the results of the comparison of the doses measured by SRSMC, the Exradin A16 microchamber, the Exradin A16 microchamber with the applied field output correction factors of TRS‐483, and the dose calculated by TPS. The dose errors of SRSMC compared to those of the Exradin A16 microchamber and TPS tended to increase in an irradiation field smaller than 15 mm. The maximum dose error was −11.0% in a 5‐mm field for the Exradin A16 microchamber, −1.6% in a 10‐mm field for the Exradin A16 microchamber with the applied TRS‐483 corrections, and −4.9% in a 7.5‐mm field for the TPS calculations. In irradiation fields of 12.5 mm or larger, the dose measured by SRSMC agreed with that measured by the Exradin A16 microchamber (with and without the applied TRS‐483 corrections) and calculated by TPS within 3.0%.

### 3.2 Angular dependence

**FIGURE 5:**
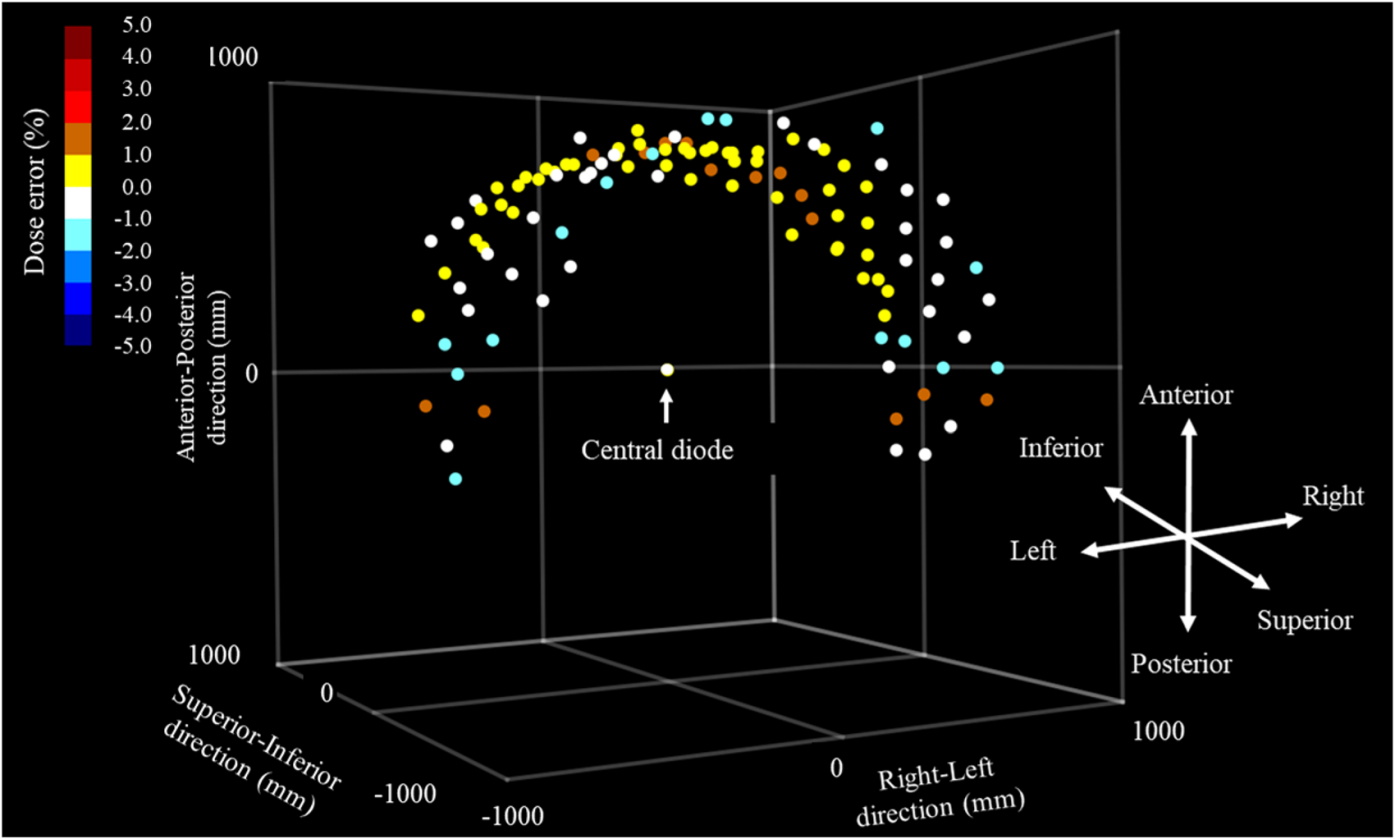
Angular dependence of SRS MapCHECK (SRSMC). The coordinates in the graph show the robot coordinates in the CyberKnife (CK). Each point shows the node coordinates placed around the central diode detector. The color bar in the upper left corner indicates the measured dose error (in %) of SRSMC relative to the measurement of the Exradin A16 microchamber

Figure 5 shows the results of the measured dose errors between SRSMC and the Exradin A16 microchamber at each node position. The measured doses of SRSMC and the Exradin A16 microchamber agreed within 2.0% for all nodes, and the mean percentage dose error for all nodes was 0.68% ± 0.45%. The largest dose error was 1.9%, corresponding to a beam angle of 6° with respect to the horizontal.

### 3.3 Absolute dose verification by the high‐resolution 2D detector‐based dose verification system

Table 2 shows the results of the absolute dose verification experiments. For P0, the dose errors of SRSMC with respect to the calculated doses of FSPB+ and MC were −0.6% and 3.2%, respectively, and −0.1% with respect to the measured doses of the Exradin A16 microchamber. By contrast, for P1, P2, and P3, the dose errors of SRSMC relative to the TPS were −0.4%, −0.1%, and 0.0% for FSPB+; 4.0%, 4.2%, and 4.0% for MC; and −1.4%, −1.7%, and −0.9% for the Exradin A16 microchamber, respectively. The doses measured by SRSMC agreed with those of FSPB+ and the Exradin A16 microchamber within 2.0%. However, slightly higher dose errors were observed when MC was used.

**TABLE 2:** Calculated (treatment planning system [TPS]) and measured (A16 and SRS MapCHECK [SRSMC]) doses and dose errors

		Calculated or measured dose (Gy)			Dose error (%)	
Plan	Algorithm	TPS	A16	SRSMC	SRSMC versus TPS	SRSMC versus A16
P0	FSPB+	15.28	15.21	15.20	−0.6	−0.1
	MC	14.73			3.2	
P1	FSPB+	14.89	15.04	14.83	−0.4	−1.4
	MC	14.26			4.0	
P2	FSPB+	15.00	15.23	14.98	−0.1	−1.7
	MC	14.38			4.2	
P3	FSPB+	15.29	15.43	15.29	0.0	−0.9
	MC	14.70			4.0	

Abbreviations: FSPB, finite‐size pencil beam; MC, Monte Carlo; SRSMC, SRS MapCHECK; TPS, treatment planning system.

### 3.4 Dose distribution verification using the high‐resolution 2D detector‐based dose verification system

**FIGURE 6:**
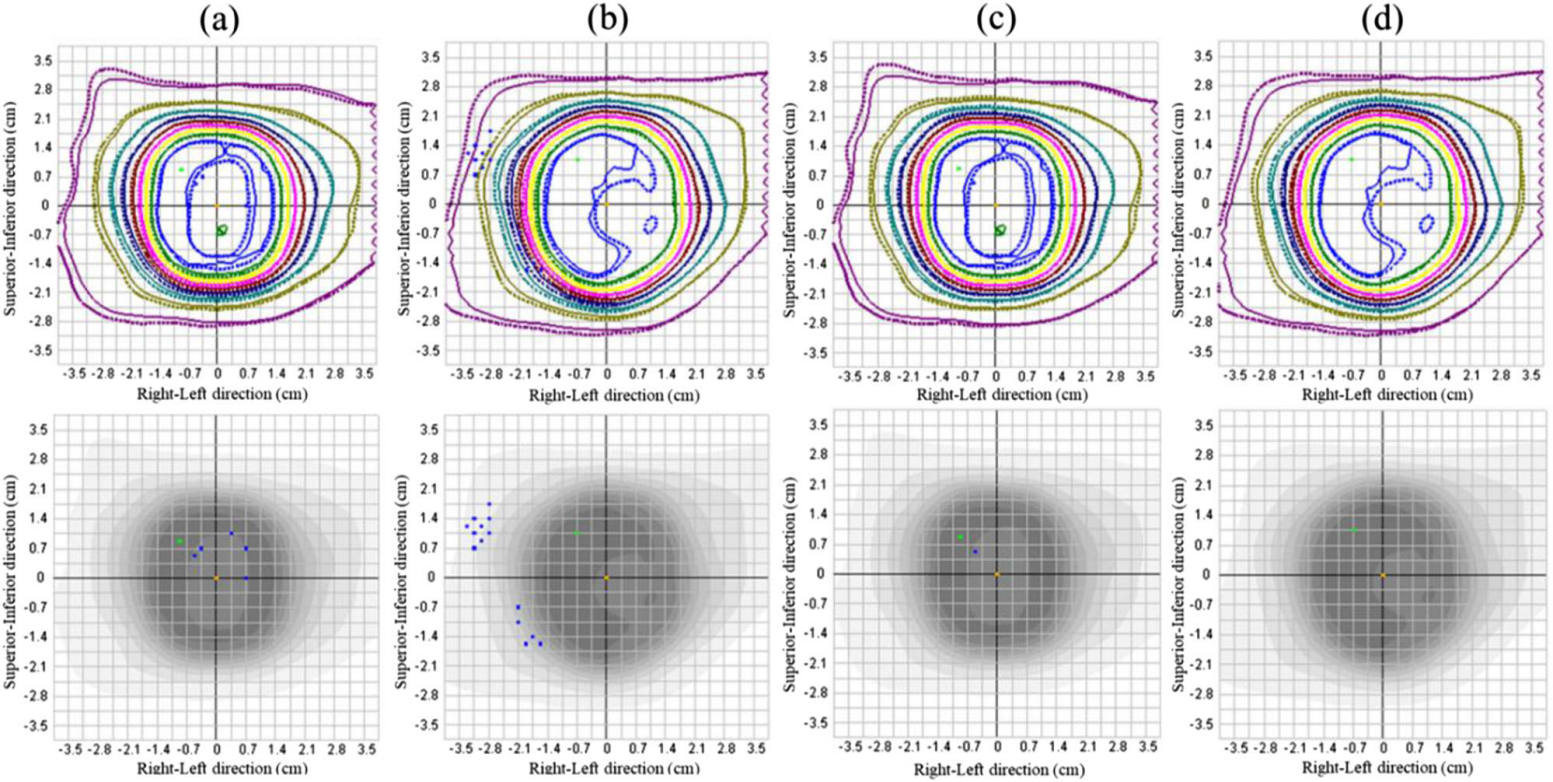
Dose distribution analysis of SRS MapCHECK (SRSMC). (a) SRSMC versus FSPB+ dose (P0, absolute position), (b) SRSMC versus FSPB+ dose (P2, absolute position), (c) SRSMC versus FSPB+ dose (P0, relative position), and (d) SRSMC versus FSPB+ dose (P2, relative position). The upper panel shows the isodose overlay. The solid lines indicate the isodose line measured by SRSMC, whereas the dotted lines indicate the isodose line calculated by the treatment planning system (TPS). The lower panel shows the gamma analysis results. The blue dots show failure sections in the gamma analysis

An example of the comparisons of the dose distributions between SRSMC and TPS calculation (FSPB+ algorithm) under the absolute or relative position analyses is shown in Figure 6. The results of the comparison under the absolute position analysis showed that the dose distributions of SRSMC tended to shift to the left‐hand and superior sides of the patient in all plans. The slight positional corrections for the calculated dose distribution led to the reduction of the disagreement between the dose distributions, and the gamma pass rates were increased in all plans.

**FIGURE 7:**
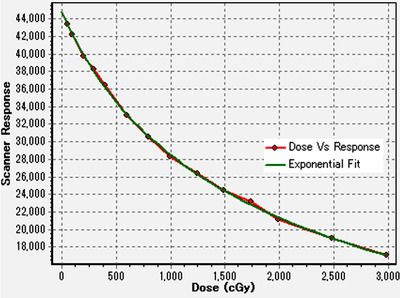
Dose calibration curve for a GAFchromic EBT‐XD film created in the SNC Patient software. The dot marks with red line represent the scanned data, and the green exponential fit curve was used for the conversions from the scanner response values to the doses for the dose distribution verification

**FIGURE 8:**
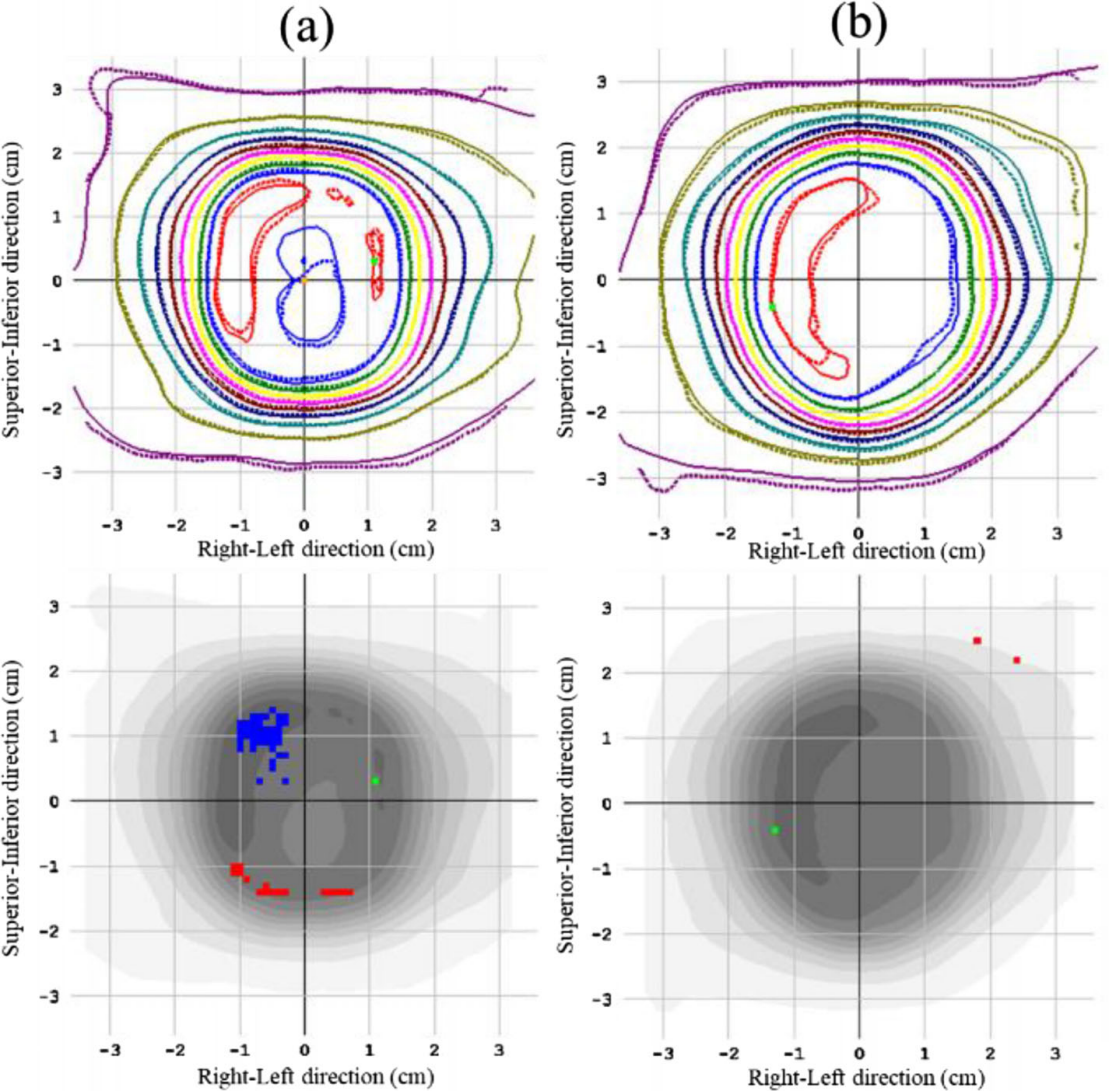
Dose distribution analysis of SRS MapCHECK (SRSMC): (a) SRSMC versus film (P0, relative position) and (b) SRSMC versus film (P2, relative position). The upper panel shows the isodose overlay. The solid lines indicate the isodose line measured by SRSMC, whereas the dotted lines indicate the isodose line measured by the film. The lower panel shows the gamma analysis results. The red and blue dotted areas show failure sections in the gamma analysis

The dose calibration curve for the dose distribution verification using the radiochromic film is shown in Figure 7. The exponential fit curve was used for the conversions from the scanner response values to the doses. Figure 8 shows an example of the comparison of the dose distributions between SRSMC and EBT‐XD film for the same motion conditions as mentioned previously.

Table 3 summarizes the results of the dose distribution verification experiments. As shown in this table, the gamma pass rates of SRSMC compared to the calculated doses of FSPB+ and MC under the P0 condition were 99.3% for both absolute positions and 99.8% and 99.4% for the relative positions, respectively. By contrast, the gamma pass rates for P1, P2, and P3 were 99.7%, 98.0%, and 100% for FSPB+ and 98.8%, 93.5%, and 97.2% for MC, respectively. In the evaluation of the relative position, the gamma pass rate was greater than 95% in all plans. Considering the comparison between SRSMC and the EBT‐XD film, the gamma pass rates were greater than 95% in all plans.

**TABLE 3:** Gamma pass rates between the dose distributions obtained by SRS MapCHECK (SRSMC) and treatment planning system (TPS) or EBT‐XD film

		Versus TPS (coronal)		
Plan	Algorithm	Absolute position	Relative position	Versus film Relative position
P0	FSPB+	99.3	99.8	97.9
	MC	99.3	99.4	
P1	FSPB+	99.7	99.8	99.2
	MC	98.8	99.2	
P2	FSPB+	98.0	100.0	99.0
	MC	93.5	98.3	
P3	FSPB+	100.0	100.0	99.4
	MC	97.2	97.9	

Abbreviations: FSPB, finite‐size pencil beam; MC, Monte Carlo; TPS, treatment planning system.

## 4 DISCUSSION

In this study, we evaluated the basic dosimetric characteristics of SRSMC for CK. As CK uses many small irradiation fields, it is important to evaluate the response of SRSMC to small irradiation fields. Therefore, in this study, the field size dependence of SRSMC for CK was evaluated by comparing its OPF with that of the microDiamond detector. We selected the microDiamond detector for comparison because it has been reported that this detector is suitable for the measurement of small irradiation fields considering dosimetric characterizations such as the field size dependence.^28^, ^29^ In addition, the field output correction factor of the microDiamond detector described in TRS‐483, which is a guideline for the measurement of small irradiation fields, was incorporated for the OPF and absolute dose for the field size dependence evaluations conducted in this study.

In a previous report by Ahmed et al.,^15^ the OPF of SRSMC for the general‐purpose linac tended to be ∼0.0%–2.5% lower than that of the W1‐plastic scintillation detector (W1‐PSD) in irradiation fields of 10 × 10 to 40 × 40 mm^2^, whereas it was ∼0.5%–3.2% higher in the smallest irradiation field of 5 × 5 mm^2^. In this study, the same tendency was observed except for the smallest irradiation field of a 5‐mm diameter in the fixed circular collimator of CK. For the 5‐mm diameter, the OPF of SRSMC for CK was consistent with that of the microDiamond detector. We consider that the difference between the results of Ahmed et al. and this study in the small irradiation fields is due to the differences in collimator structure, beam quality, and the dosimetric characterizations of the detector. Moreover, it was reported that the microDiamond detector demonstrated an ∼3.0%–5.0% larger response than that of W1‐PSD in a 5 × 5‐mm^2^ irradiation field of the general‐purpose linac in a previous study.^30^

In the evaluation of the absolute dose for the SRSMC irradiation field size, the doses measured by SRSMC were compared with that measured by the Exradin A16 microchamber and that calculated by the TPS. The Exradin A16 microchamber is mainly used in patient‐specific absolute dose verification at our institute; thus, we chose it for comparison. The dose measured by SRSMC tended to be higher than that of the Exradin A16 microchamber and smaller than that of TPS in irradiation fields of 15 mm or smaller. This tendency between SRSMC and the Exradin A16 microchamber in the small field was consistent with a previous report that the Exradin A16 microchamber tended to provide decreased results in response to small irradiation fields.^31^ The dose errors of SRSMC were reduced by applying the field output correction factor of TRS‐483. However, for a 5‐mm field diameter with the Exradin A16 microchamber, a field output correction factor was not provided by TRS‐483; therefore, these data were not plotted in Figure 4. Frequently, 10‐mm or smaller fixed circular collimators are used for treatment planning for cerebral arteriovenous malformations and trigeminal neuralgia in CK.^32^, ^33^ The disagreements in the absolute doses between SRSMC and TPS calculation in the small fields were relatively larger than those for a field size of 12.5 mm or larger and may require careful consideration in the dose verification for treatment planning using many small fields.

In the measurement of angular dependence, the dose errors between SRSMC and the Exradin A16 microchamber were within 2.0% for all nodes. In particular, the dose errors were larger for the nodes that were incident at an angle of 10° or less with respect to the horizontal plane for SRSMC, exhibiting the same tendency reported by Ahmed et al.^15^ In this study, the maximum dose error (1.9%) was smaller than that reported by Ahmed et al. (at ∼3.0%). The tendency for the response of SRSMC to decrease with the horizontal incidence and increase with the incidence from below was also similar to that reported by Ahmed et al. Such angular dependence also exists in the other two‐dimensional detectors, but the effect on the composite measurement results has been reported to be small.^34^, ^35^ In addition, as CK performs extremely multiportal irradiation, the effect of the incidence angle for each beam may be dispersed.

In this study, dose verification was performed using the treatment plan used in a previous report by Kawata et al.^7^ The gamma pass rates in this study were higher than those reported by Kawata et al. for all plans. Kawata et al. performed irradiation using an inhomogeneous moving thorax phantom (Model 008A; CIRS Inc., Norfolk, VA, USA). However, StereoPHAN is a homogeneous structure, and this difference in the phantom structure might have affected the obtained results. The absolute dose verification results of SRSMC in the tumor‐tracking irradiation verification system showed larger errors during the moving conditions than in the static condition when compared to the error of the TPS calculation (FSPB+ algorism) and Exradin A16 microchamber. This tendency is consistent with the results reported by Kawata et al. A multi‐institute study comparing the dose distribution of SRSMC in the static condition with that of the TPS and films in a general‐purpose linac was performed in a previous study by Rose et al.^16^ Gamma pass rates greater than 90% were considered to be passing, and 89% and 95% of cases exceeded the threshold in the comparison with the TPS and film results, respectively. In this study, the gamma pass rates exceeded 95% for all plans when comparing the doses measured by SRSMC and the doses calculated by the TPS using the FSPB+ algorithm. In addition, the gamma pass rates exceeded 95% for all plans in the comparison of the SRSMC and film doses. Therefore, SRSMC can be used for a dose verification of tumor‐tracking irradiation for CK, which is equivalent to the general‐purpose linac. However, we evaluated a moving platform, and the respiratory motion was limited to the S–I direction. In addition, actual patient respiratory waveforms and hysteresis movements have not yet been evaluated.^36^ This should be investigated in future studies.

The SRSMC and StereoPHAN phantom regions were overwritten with a mass density of 1.2 g/cm^3^ for the calculations using MC in this study. When using the MC algorithm implemented in Precision TPS, each voxel is assigned to one of three material types based on its mass density^23^: air, soft tissue, or bone. The material type is used to define the photon mean free path within each material type at a reference density as a function of the photon energy. The mass density boundary between the soft tissue and bone was 1.125 g/cm^3^. The StereoPHAN phantom region is recognized as a bone material type, and the mean free path of the bone material type is used for the calculation. We consider that this limitation of the dose calculation algorithm will increase the uncertainty in dose verification using the StereoPHAN phantom. Therefore, absolute dose calibrations for the field size dependence were performed using the FSPB+ algorithm. Moreover, the dose errors for MC in the tumor‐tracking dose verifications were relatively larger than those of FSPB+. We considered that this was due to a combination of the limitations of the material assignment for the MC calculation algorithm and the initial dosimetric commissioning of the TPS. The use of MC for dose verification needs to be further considered in future studies.

When using radiochromic films for dose or dose distribution verification, it is essential to obtain the dose calibration curve in advance, and a complicated analysis procedure (i.e., scanning the irradiated films a few hours after irradiation and transferring the scanned data to the analyzing software) is necessary.^37^ These procedures require a significant amount of time. In addition, it is necessary to consider the uncertainty derived from the characterizations of the film and scanner.^38^, ^39^, ^40^ However, the tumor‐tracking dose verification system using SRSMC is very easy to construct because it can be developed by simply placing SRSMC on the moving platform. Once the detector is calibrated, there is no need for complicated procedures for the next process, and the verification results can be checked immediately after irradiation is completed. In addition, no costs are required for consumables such as films. Compared to the conventional method utilizing films, the tumor‐tracking dose verification system investigated in this study can perform measurements simply, quickly, and efficiently and provides results equivalent to those of conventional methods.

## 5 CONCLUSION

In this study, we evaluated the basic characteristics of SRSMC for CK and the possibility of applying this system to tumor‐tracking dose verification. To achieve the basic characterization of SRSMC for CK, the field size dependence was evaluated as <±3.0% for almost all field sizes, whereas the angular dependence was <±2.0% for all incidence angles. These were determined to be acceptable results for clinical use for CK. However, the dose errors in the field size dependence evaluation between SRSMC and TPS calculation tended to increase when the field size was smaller than 10 mm; therefore, careful evaluation is required when evaluating plans that mainly utilize small irradiation fields. The results of the tumor‐tracking dose verification system of SRSMC were equivalent to those of conventional methods (i.e., using a microchamber detector or radiochromic film), and the proposed system is expected to contribute to improving the efficiency of quality control for tumor‐tracking treatments in many facilities.

## CONFLICT OF INTEREST

The authors declare that there is no conflict of interest that could be perceived as prejudicing the impartiality of the research reported.

## AUTHOR CONTRIBUTIONS

The conceptual design of the study was carried out by Fumitaka Kawabata, Takeshi Kamomae, Kuniyasu Okudaira, Masataka Komori, Hiroshi Oguchi, Motoharu Sasaki, Masaki Mori, Mariko Kawamura, Shinji Abe, Shunichi Ishihara, and Shinji Naganawa. Fumitaka Kawabata and Takeshi Kamomae wrote the main manuscript. Fumitaka Kawabata collected the data and performed the data analyses. All authors read and approved the final manuscript as submitted.

## Data Availability

The data that support the findings of this study are available from the corresponding author upon reasonable request.
